# Case report: Expanding the phenotype of *FOXP1*-related intellectual disability syndrome and hyperkinetic movement disorder in differential diagnosis with epileptic seizures

**DOI:** 10.3389/fneur.2023.1207176

**Published:** 2023-07-14

**Authors:** Carlo Alberto Cesaroni, Marzia Pollazzon, Cecilia Mancini, Susanna Rizzi, Camilla Cappelletti, Simone Pizzi, Daniele Frattini, Carlotta Spagnoli, Stefano Giuseppe Caraffi, Roberta Zuntini, Gabriele Trimarchi, Marcello Niceta, Francesca Clementina Radio, Marco Tartaglia, Livia Garavelli, Carlo Fusco

**Affiliations:** ^1^Child Neurology and Psychiatry Unit, Pediatric Neurophysiology Laboratory, Mother-Child Department, Azienda USL-IRCCS Di Reggio Emilia, Reggio Emilia, Italy; ^2^Medical Genetics Unit, Mother-Child Department, Azienda USL-IRCCS of Reggio Emilia, Reggio Emilia, Italy; ^3^Molecular Genetics and Functional Genomics Unit, Ospedale Pediatrico Bambino Gesù, IRCCS, Rome, Italy

**Keywords:** autism, epilepsy, movement disorder, angiomas, choanal atresia, *FOXP1*

## Abstract

**Objective:**

We aimed to report on previously unappreciated clinical features associated with *FOXP1*-related intellectual disability (ID) syndrome, a rare neurodevelopmental disorder characterized by global developmental delay, intellectual disability, and language delay, with or without autistic features.

**Methods:**

We performed whole-exome sequencing (WES) to molecularly characterize an individual presenting with ID, epilepsy, autism spectrum disorder, behavioral problems, and facial dysmorphisms as major features.

**Results:**

WES allowed us to identify a previously unreported *de novo* splice site variant, c.1429-1G>T (NM_032682.6), in the *FOXP1* gene (OMIM^*^605515) as the causative event underlying the phenotype. Clinical reassessment of the patient and revision of the literature allowed us to refine the phenotype associated with *FOXP1* haploinsufficiency, including hyperkinetic movement disorder and flat angiomas as associated features. Interestingly, the patient also has an asymmetric face and choanal atresia and a novel *de novo* variant of the *CHD7* gene.

**Conclusion:**

We suggest that *FOXP1*-related ID syndrome may also predispose to the development of hyperkinetic movement disorders and flat angiomas. These features could therefore require specific management of this condition.

## 1. Introduction

The *FOXP1* (forkhead box P1) gene (MIM ^*^605515) codifies for a member of the forkhead box family of transcriptional factors playing important roles in the regulation of gene transcription from early development through adulthood (1, 2). The identification of deletions encompassing *FOXP1* (3–11) and loss-of-function variants of this gene in individuals with a consistent clinical phenotype (12–20) has led to the conclusion that *FOXP1* haploinsufficiency is the molecular basis for the core features of “*FOXP1*-related intellectual disability (ID) syndrome” (MIM #613670). In addition to ID (mostly mild to moderate), the clinical phenotype of this condition includes a global developmental delay mainly affecting language, autistic spectrum disorder (ASD), behavioral disorders, facial dysmorphisms, ophthalmological anomalies, and cardiac and genitourinary anomalies (21–23). Epilepsy and kidney ailments (unilateral renal agenesis and horseshoe kidney) have been reported in some instances (20, 24). Approximately 100 cases have been described, and in the cases in which the segregation analysis was performed, the disease-causing variant was found to be *de novo*.

Herein, we report on a patient with a *de novo* splice site variant in *FOXP1, NM_032682.6:*c.1429-1G>T, identified by means of whole-exome sequencing (WES) and who has a complex phenotype with features that had not previously been described in this disorder. The boy has a psychomotor delay, ID, epilepsy, ASD, behavioral problems, and dysmorphic features. At birth, he also showed flat angiomas and right choanal atresia. There is no family history of similar features. Moreover, since the age of 9 years, he has been showing a stereotyped hyperkinetic movement disorder not associated with EEG anomalies. While a *de novo* variant in *CHD7*, NM_032682.6:c.122T>C, p.(Met41Thr) could explain the right choanal atresia, flat angiomas and the movement disorder are likely to represent new features of this condition that had not previously been characterized.

## 2. Case report

### 2.1. Medical history

The patient, a 12-year-old boy, is the second child of apparently healthy and unrelated parents. The older sister of the boy is healthy. The second pregnancy of the parents terminated in a miscarriage. Their family history is negative for motor tics. Pregnancy was complicated by gestational diabetes and preeclampsia. The proband was born at 36 weeks of gestation by vaginal delivery. Birth parameters were a weight of 3,200 g (75th−90th percentile), a length of 51 cm (90th−97th percentile), and a head circumference of 36 cm (98th percentile). The Apgar scores were 9 and 9 at 1 and 5 min, respectively. He was admitted to the Neonatal Intensive Care Unit (NICU) for approximately 2 h for cyanosis. During investigations, right choanal atresia was discovered, with subsequent daytime apneas. He underwent surgery for left cryptorchidism at the ages of 3, 4, and 10 years. At 10 years, he underwent permanent Appendix vesicostomy for urinary incontinence. He showed psychomotor delay: walked on tiptoes with help at 17 months; said first words at 4 years of age; and showed partial sphincter control at 4 years, with enuresis. At the age of 12 years, he was able to pronounce sentences but he could not eat on his own. He showed talipes equinovarus with persistent tiptoe walking and had motor impairment with difficulty in independent ambulation. Braces on the lower limbs and wheelchairs for long journeys were introduced.

**Figure 1:**
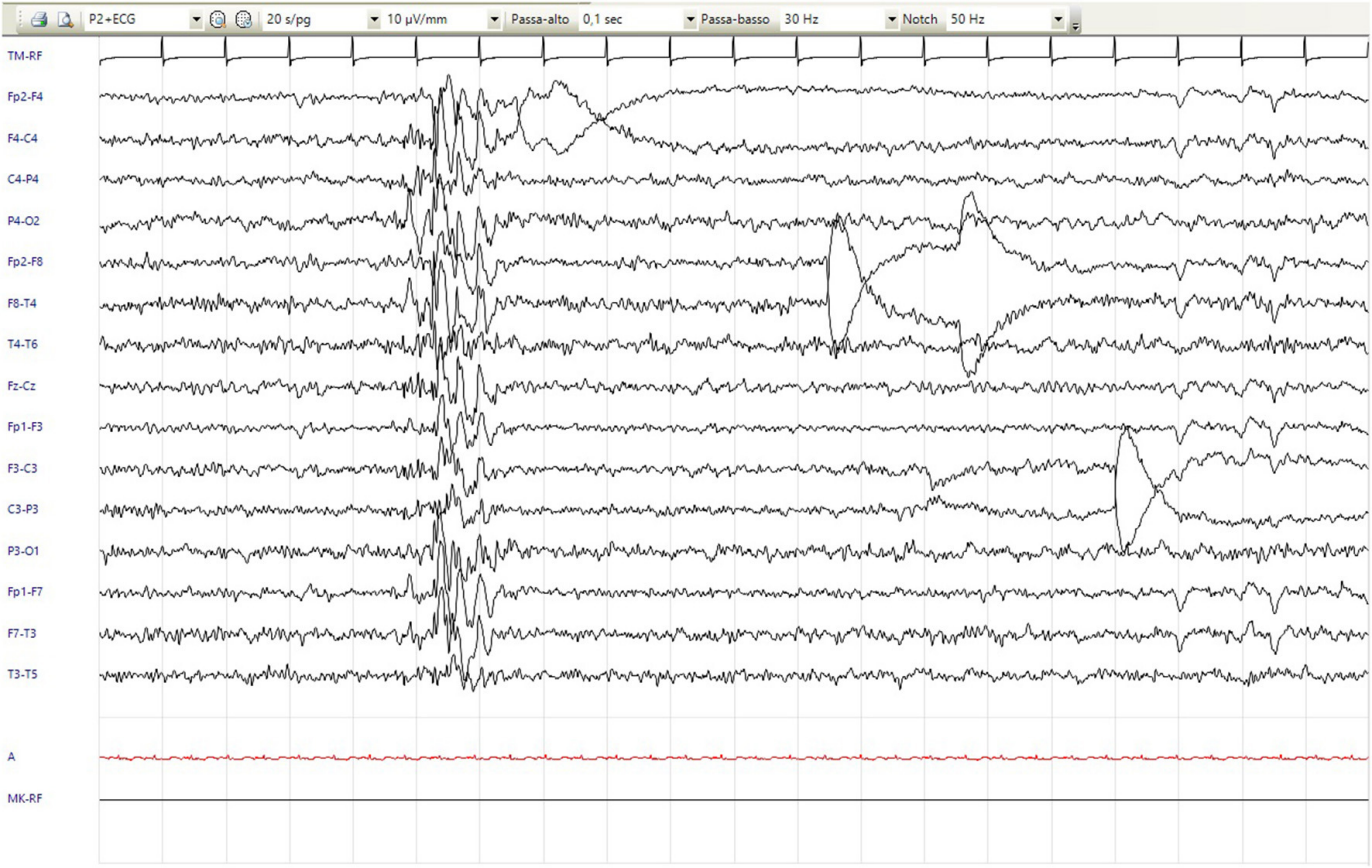
EEG showed short diffuse bursts of epileptic anomalies (20/s/pg, 10 μV/mm, 0,1 s, 30 Hz).

Motor stereotypies with rocking of the trunk appeared at 7 years. Since the age of 8 years, he has shown recurrent stereotyped hyperkinetic movements, including retrocollis with tongue protrusion and hypertonicity of the arms, that repeat a few times in a less intense fashion and last up to 2 min, which is definable as paroxysmal dystonia (Supplementary Video S1). Sometimes, these episodes were associated with apparent alteration of contact with fixed-gaze focal abnormalities, and bouffées of epileptiform anomalies were recorded. Although not concomitant with these episodes, therapy with valproic acid and clobazam was subsequently introduced, with no benefit. Follow-up routine EEG showed focal paroxysmal anomalies in the phase of reduced alertness and short diffuse bursts of epileptic abnormalities (Figure 1). Since the age of 11 years, a disinhibited conduct of self-stimulation and autoeroticism appeared and required the introduction of risperidone. Hetero-aggressivity and hand stereotypies (previously rubbing and recently clapping) were reported. We also noticed a sensory-seeking behavior with a tendency to seek close physical contact and to smell people, hands, and food.

### 2.2. Clinical and instrumental findings

**Figure 2:**
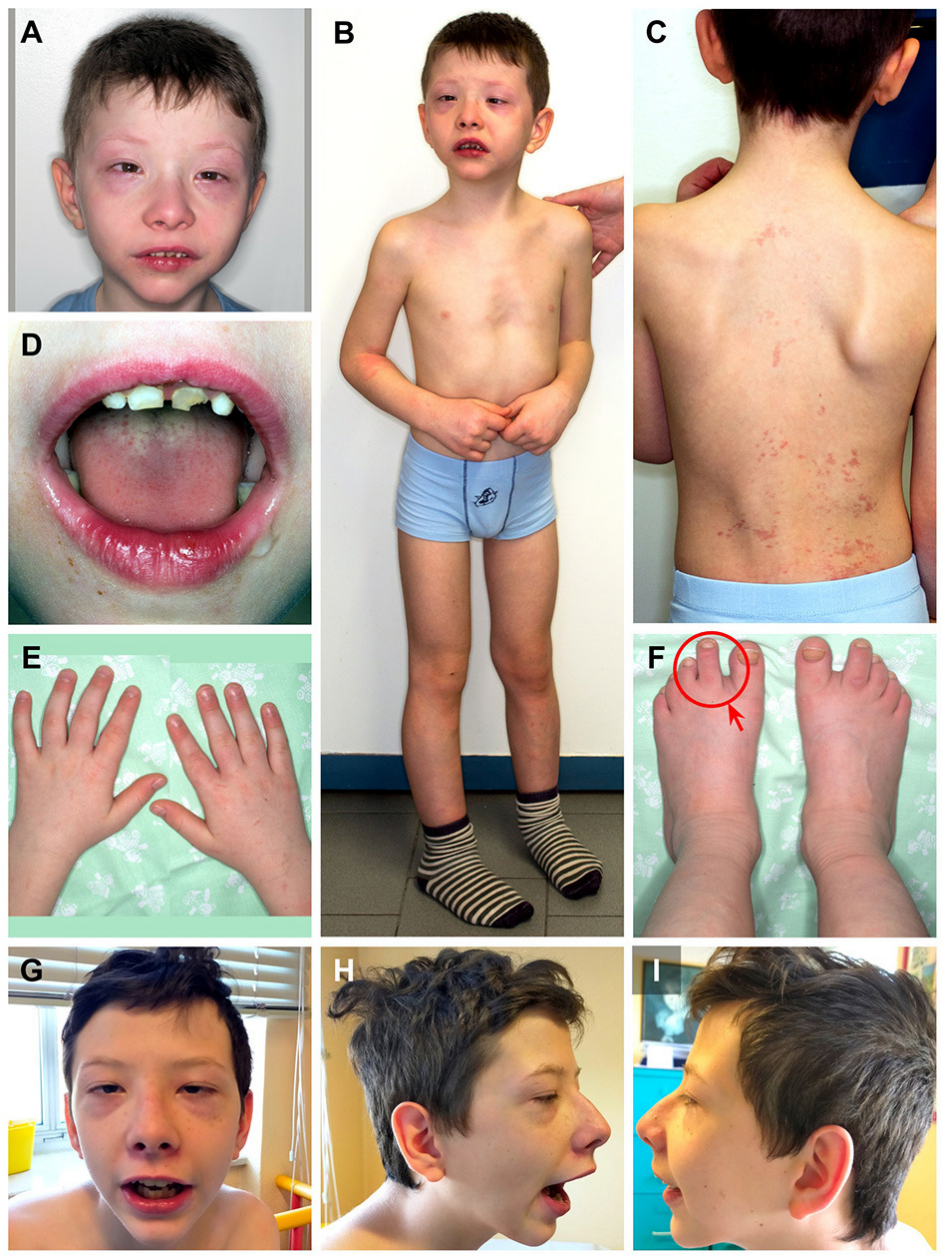
Patient at 9 years and 8 months of age **(A-F)**. **(A)** Hypertelorism, periorbital fullness, epicanthus, esotropia, thick alae nasi, and microretrognathia; **(B)** pectus excavatum; **(C)** scoliosis, congenital vascular anomalies that disappeared upon pressure in the occiput, vertex, and multiple in the back; **(D)** small and spaced teeth; **(E)** widening of the proximal interphalangeal joints of the hands; and **(F)** flat feet with sandal gap and II-III toe partial cutaneous syndactyly (red circle and arrow). Patient at 12 years and 6 months of age **(G–I)**. Note the more pronounced nose and microretrognathia.

**Figure 3:**
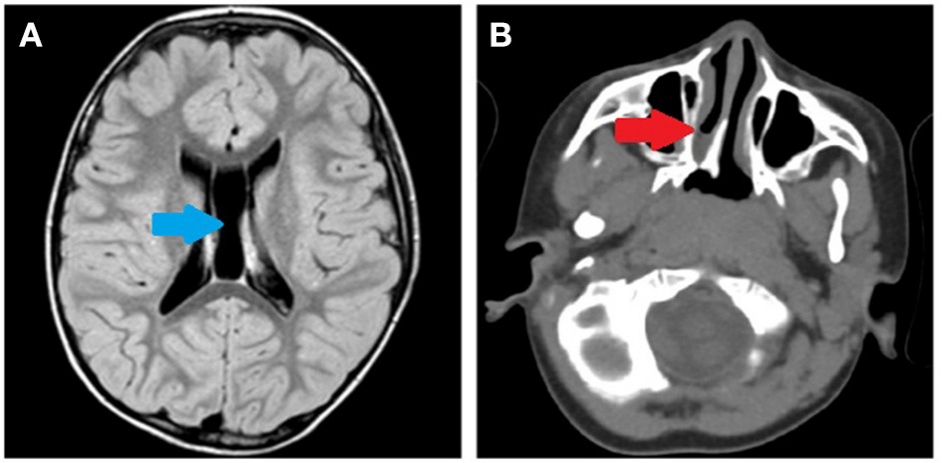
**(A)** Brain MRI performed at 6 years of age. Cysts of the septum pellucidum and the cavum vergae (blue arrow). **(B)** Brain CT scan performed at 3 years of age. Right choanal atresia (red arrow).

During clinical follow-up, a progressively more elongated face and a more pronounced nasal bridge were noticed (Figure 2). At the last examination (12 years and 6 months), his height was 163.5 cm (>97th percentile), weight was 45 kg (75th−90th percentile), and head circumference was 54 cm (50th−75th percentile). He had a hypomimic and asymmetric face, flat occiput, hypertelorism, periorbital fullness, bilateral ptosis (more pronounced on the left side), epicanthus, exotropia, thick alae nasi, microretrognathia, a thin upper lip, a high-arched palate, small and spaced teeth, poor representation of muscle mass, pectus excavatum, scoliosis, widening of the proximal interphalangeal joints of the hands, flat feet with sandal gap, and II-III toe partial cutaneous syndactyly. He also showed vascular anomalies: visible vessels on the lower eyelids and congenital vascular anomalies (flat angiomas) that disappeared upon pressure, some in the occiput and vertex, and multiple in the back (Figure 2). Abdominal ultrasound, audiometric testing, fundus examination, and electroneurography (ENG) were normal. A small aneurysm at the level of the interatrial septum was detected during the echocardiography. Brain MRI at 6 years revealed cysts of the septum pellucidum and of the *cavum vergae* (Figure 3A). A brain CT scan performed at 3 years of age revealed right choanal atresia (Figure 3B). The ophthalmological evaluation documented hyperopia and astigmatism.

### 2.3. Molecular analysis

Genetic works based on CGH array and *FMR1* analyses revealed normal results. WES analysis was performed on genomic DNA extracted from peripheral blood leukocytes of the proband and their parents under standard procedures. The capture of the target regions was performed using the SureSelect Human All Exon V7 target enrichment kit, and sequencing was performed on a NovaSeq 6000 platform (Supplementary Table S1). WES raw data were processed using an in-house pipeline based on the GATK Best Practices (25–28). The UCSC GRCh37/hg19 genome assembly was used as a reference for reads alignment by means of the BWA-MEM (bwakit v.0.7.11) algorithm (29). SnpEff v.5.0 (30) and dbNSFP v.4.0 (31) tools were used for variant annotation, including functional impact and *in silico* prediction of deleteriousness by means of Combined Annotation Dependent Depletion (CADD) v.1.4 (32), Mendelian Clinically Applicable Pathogenicity (M-CAP) v.1.3 (33), and Intervar v.2.0.1 (34). Population frequencies were annotated from the gnomAD database and an in-house database, which included ~2,500 exomes. High-quality SNV and INDELs (GATK v3.8 hard-filtering: QUAL>100) with low-frequency (gnomAD MAF <0.1%, in-house exomes database <1) and within coding exons and splice regions were assessed separately. In the functional impact evaluation, a higher priority was assigned to variants with a CADD score of >20, was tagged as pathogenic/likely pathogenic by InterVar, and relied on Phen2Gene, a tool dedicated to the gene/phenotype associations (35). WES analysis led to the identification of two possibly relevant *de novo* variants: a pathogenic splice donor site change involving the *FOXP1* gene [NM_032682.6:c.1429-1G>T] and a missense variant in *CHD7* gene (NM_017780.4:c.122T>C), which predicted to result in the amino acid substitution p.(Met41Thr). Both variants have been submitted to ClinVar (SUB13065401). The *FOXP1* variant is absent in the reference population database gnomAD v2.1.1 (accessed on 2023/02/28) and is predicted to abolish the canonical acceptor splice site of exon 17, possibly activating a nearby cryptic acceptor site downstream that would cause a frameshift and generate a premature stop codon. According to the recommendations of the American College of Medical Genetics and Genomics (36, 37), in the absence of experimental data on the effects of the splice site alteration, the variant can be classified as likely pathogenic. The *CHD7* variant is reported at an extremely low frequency in the reference population database gnomAD v2.1.1 (accessed on 2023/02/28), predicted damaging by most *in silico* tools, and can be classified as a variant of uncertain significance (VUS) favoring likely pathogenic, possibly contributing to a subset of the reported clinical features (i.e., asymmetric face and monolateral choanal atresia).

## 3. Discussion

Our patient had “*FOXP1*-related intellectual disability (ID) syndrome” with a clinical phenotype fitting the condition with many features that had already been described in previous reports. He presented with mild/moderate ID, a speech delay with echolalia, autism spectrum disorder features, and dysmorphisms. Hyperopia and astigmatism had also been reported. On the contrary, cardiac defects are minor and often restricted to atrial septal defects (38). Notably, Trelles et al. (39) described the complex neurobehavioral profile of the condition. This includes repetitive behaviors and sensory symptoms (e.g., sensory seeking), some of which were also recognized in our patient. Compared to the other described cases, however, the patient also had peculiar characteristics; in particular, he showed hyperkinetic movement disorder not associated with electroencephalographic anomalies and he also had EEG anomalies without clinical manifestations. Therefore, we cannot rule out both a movement disorder and seizures. Among almost 100 individuals with *FOXP1*-related ID syndrome, seven individuals experienced epilepsy, but no subjects have been reported to exhibit movement disorder (Supplementary Table S2), suggesting another neurological feature that might occur in this condition. A more extended series of cases, however, is required to corroborate this assumption.

The FOXP1 transcriptional repressor is a molecular determinant of radial neuronal migration and morphogenesis (40). In particular, *Foxp1* is expressed at high levels in a subset of striatal projection neurons of the mouse brain, probably the matrix neurons (41, 42). *Foxp1* is crucial for maintaining the cellular composition of the striatum and the proper formation of the striosome-matrix compartments (43). Moreover, gene expression studies in human neural progenitors with altered *FOXP1* levels documented a role in regulating pathways involved in striatal neuron identity (44). The expression pattern of *Foxp1* mRNA suggests that Foxp1 may play a role in the development and formation of a circuit in the basal ganglia. A similar mechanism is described for Huntington's disease, in which a reduced expression of *Foxp1* correlates with the drop of the neuroprotective role in the affected cells (45). Though we have not demonstrated this in our patient, we can speculate that the possible alterations of the mRNA in the basal ganglia could possibly explain his movement disorder (41). In addition, mice heterozygous for a *Foxp1* deletion (46) exhibit a reduced mitochondrial membrane potential and complex I activity, as well as decreased expression of the antioxidants superoxide dismutase 2 and glutathione peroxidase 1, resulting in increased oxidative stress and lipid peroxidation. Note that a mitochondrial disorder involving oxidative-stress-related *SUCLA*2 gene is associated with a hyperkinetic movement disorder (47). The presence of multiple flat angiomas is also peculiar considering that cutaneous vascular anomalies are unexpected in the disorder. Using gene expression profiling, Grundmann et al. (48) showed that *FoxP1* induces a specific change in the endothelial transcriptome and stimulates angiogenesis by repressing semaphorin 5B (Sema5B). We, therefore, suggest that the congenital vascular anomalies (flat angiomas) observed in our patient could also belong to the *FOXP1*-related spectrum.

The present subject also has right choanal atresia and facial asymmetry, which have never been described in the *FOXP1*-related ID syndrome. We can speculate that these features may be related to the *de novo* p.(Met41Thr) variant in the *CHD7* gene. This gene is usually involved in CHARGE syndrome, and we cannot exclude the contribution of this variant to the complex phenotype of the present subject.

In conclusion, the features that we have described and that distinguish our patient broaden the phenotype of *FOXP1*-related syndrome and can contribute to defining a more specific follow-up of the patient.

## Data availability statement

The datasets presented in this study can be found in online repositories. The names of the repository/repositories and accession number(s) can be found here: https://www.ncbi.nlm.nih.gov/clinvar/, SUB13065401.

## Ethics statement

The studies involving human participants were reviewed and approved by the Local Institutional Ethical Committee of the Ospedale Pediatrico Bambino Gesù IRCCS (OPBG), Rome (1702_OPBG_2018). Written informed consent to participate in this study was provided by the participants' legal guardian/next of kin. Written informed consent was obtained from the individual(s), and minor(s)' legal guardian/next of kin, for the publication of any potentially identifiable images or data included in this article.

## Author contributions

CAC and MP: conceptualization and writing—original draft preparation. CM, CAC, SP, MN, FCR, MT, SGC, and RZ: genomic analyses. CAC, MP, GT, SR, DF, and CS: clinical data collection and data curation. CAC, MP, SP, FCR, MT, SGC, and RZ: writing—review and editing. LG and CF: supervision. All authors have read and agreed to the published version of the manuscript.
