# Supplementary material for: Case report: Expanding the phenotype of FOXP1-related intellectual disability syndrome and hyperkinetic movement disorder in differential diagnosis with epileptic seizures
Source: Front Neurol. 2023 Jul 14;14:1207176. doi: 10.3389/fneur.2023.1207176 (PMC10382204; doi:10.3389/fneur.2023.1207176)
Supplement: Supplementary file 1 [file Table_1.docx]

| **Neurological features** | Pariani et al. (2009) | Carr et al. (2010) | Ţuţulan-Cunită et al. (2012) | Palumbo et al. (2013) | Le Fevre et al. (2013) | Dimitrov et al. (2015) | Sollis et al. (2017) | Siper et al. (2017) | Meerschaut et al. (2017) | Johnson et al. (2018) | Yamamoto et al. (2019) | Vuillaume et al. (2018) | Urreizti et al. (2018) | Mutlu-Albayrak et al. (2019) | **Our patient** | **Total** |
| --- | --- | --- | --- | --- | --- | --- | --- | --- | --- | --- | --- | --- | --- | --- | --- | --- |
| Number of examined patients | 1 | 1 | 1 | 1 | 1 | 3 | 3 | 9 | 23 | 1 | 3 | 1 | 1 | 1 | **1** | **51** |
| Spinal defects | N.A. | N.A. | 1/1 | N.A. | N.A. | N.A. | N.A. | 1/9 | 2/23 | N.A. | N.A. | N.A. | N.A. | N.A. | **1/1** | **5/34 (15%)** |
| Hypertonia | 1/1 | 1/1 | N.A. | N.A. | N.A. | 3/3 | N.A. | N.A. | 14/21 | N.A. | N.A. | N.A. | 1/1 | N.A. | N.A. | **20/27 (7%)** |
| Hyperreflexia | N.A. | 1/1 | 1/1 | N.A. | N.A. | 3/3 | N.A. | N.A. | N.A. | N.A. | N.A. | N.A. | N.A. | N.A. | N.A. | **5/5 (100%)** |
| Seizures | N.A. | 1/1 | N.A. | N.A. | N.A. | 1/3 | 1/3 | N.A. | 3/22 | N.A. | N.A. | 1/1 | N.A. | N.A. | **1/1** | **8/31 (26%)** |
| Brain abnormalities | 1/1 | 1/1 | 1/1 | N.A. | 1/1 | 3/3 | 1/2 | 6/7 | 11/23 | 1/1 | 1/3 | 1/1 | N.A. | 1/1 | **1/1** | **30/46 (65%)** |
| Abnormal EEG | N.A. | 1/1 | N.A. | 1/1 | N.A. | N.A. | N.A. | 2/4 | N.A. | N.A. | N.A. | N.A. | N.A. | N.A. | **1/1** | **5/7 (71%)** |

**Table S1. Neurological features in patients with FOXP1-related intellectual disability (ID) syndrome.**

We have reported for each study the number of examined patients (denominators) and how many patients have each feature (numerator). Unless otherwise specified in the original studies, we assume that a feature was not recorded if not reported. The penultimate column regards the neurological features of our patient. In the last column, there are the reported patients with each feature. The percentage of patients examined with the considered feature is reported in round brackets. *EEG*, electroencephalogram; *N.A.*, not applicable.
