# Supplementary material for: Case report: Expanding the phenotype of FOXP1-related intellectual disability syndrome and hyperkinetic movement disorder in differential diagnosis with epileptic seizures
Source: Front Neurol. 2023 Jul 14;14:1207176. doi: 10.3389/fneur.2023.1207176 (PMC10382204; doi:10.3389/fneur.2023.1207176)
Supplement: Supplementary file 2 [file Table_2.docx]

| **Table S2. WES statistics and data output.**   \|  \| **Pt_** \| \| --- \| --- \| \| WES enrichment kit \| SureSelect Human All Exon V7 \| \| Sequencing platform \| Illumina NovaSeq6000 \| \| Target regions coverage >10x \| 95% \| \| Target regions coverage >20x \| 93% \| \| Average depth on target \| 140x \| \| Total number of high-quality variants \| 85,863 \| \| Low frequency variants affecting CDS or splice site/regions^1^ \| 134 \| \| Putative disease associated genes^2^: \|  \| \| - candidate genes (autosomal dominant) \| 2^3^ \| \| - candidate genes (autosomal recessive/X-linked) \| 0 \| |
| --- | --- | --- | --- | --- | --- | --- | --- | --- | --- | --- | --- | --- | --- | --- | --- | --- | --- | --- | --- | --- | --- | --- |
| ^1^ High-quality, rare/private (gnomAD MAF<0.1%; in house database MAF<1%), functionally relevant variants within coding exons and splice regions (-3/+8).  ^2^ High-quality, rare/private, functionally relevant variants with CADD phred>20.0.  ^3^ *FOXP1* (c.1429-1G>T, splice acceptor site, CADD=34), *CHD7* (c.122T>C, p.Met41Thr). |
